# Single‐nucleus transcriptomics reveals transcriptional dynamics underlying maize ligule development

**DOI:** 10.1111/tpj.71194

**Published:** 2026-11-09

**Authors:** Fang Wei, Mingzhang Xia, David W. Galbraith, Jinling Huang, Chun‐Peng Song, Jinjing Pan, Xuefei Yang, Guiling Sun

**Affiliations:** ^1^ Department of Economic Plants and Biotechnology, Yunnan Key Laboratory for Wild Plant Resources, Kunming Institute of Botany Chinese Academy of Sciences Kunming 650201 China; ^2^ State Key Laboratory of Plant Diversity and Specialty Crops, Xishuangbanna Tropical Botanical Garden Chinese Academy of Sciences Kunming Yunnan 650223 China; ^3^ College of Life Sciences University of Chinese Academy of Sciences Beijing 100049 China; ^4^ School of Plant Sciences and Bio5 Institute The University of Arizona Tucson Arizona 85721 USA; ^5^ Department of Biology East Carolina University Greenville North Carolina 27858 USA; ^6^ School of Life Sciences, State Key Laboratory of Crop Stress Adaptation and Improvement, State Key Laboratory of Cotton Biology Henan University Kaifeng 475004 China; ^7^ Southeast Asia Biodiversity Research Institute Chinese Academy of Sciences Yezin Nay Pyi Taw 05282 Myanmar

**Keywords:** ligule development, single‐nucleus RNA sequencing, *ZmIDD30*, maize

## Abstract

The maize ligule arises from the preligule band (PLB) at the leaf blade‐sheath boundary and provides a tractable system for investigating how boundary‐associated positional cues direct local cell‐fate specification and tissue outgrowth. Although key genes involved in ligule development have been identified and cell‐resolved profiles are available for stages after PLB formation, how PLB acquires ligule identity and initiates outgrowth remains unclear at the cellular level. Here, we have used single‐nucleus RNA sequencing to profile maize ligular regions at the preligule band and ligule outgrowth stages. The atlas resolved major cell populations and identified a ligule population supported by established marker genes, published laser microdissection transcriptomes, and RNA *in situ* hybridization. Pseudotime trajectory and co‐expression network analyses resolved two sequential transcriptional programs corresponding to ligule initiation and subsequent outgrowth. *ZmIDD30* was identified as a promising candidate transcriptional regulator, and molecular assays showed that it bound to the promoters of *ZmPIN1c* and *ZmVG1* and activated their transcription, suggesting potential links to auxin transport and lipid metabolism during ligule development. Overall, these findings define a cell‐resolved transcriptional progression in which a localized epidermal domain at the blade‐sheath boundary progressively acquires ligule identity and progresses toward outgrowth.

## Significance Statement

How a narrow epidermal domain at the maize blade‐sheath boundary acquires ligule identity and initiates outgrowth remains poorly understood. Our snRNA‐seq atlas identifies a ligule population, resolves sequential transcriptional programs from the preligule band formation to ligule outgrowth, and identifies ZmIDD30 as a candidate regulator that directly activates the transcription of *ZmPIN1c* and *ZmVG1*, providing insight into ligule cell‐fate specification and subsequent outgrowth.

## INTRODUCTION

Plant lateral organ morphogenesis depends on the spatiotemporal establishment of developmental boundaries, the specification of localized cell fates, and their subsequent outgrowth (Zadnikova & Simon, 2014). In grass leaves, such boundaries separate adjacent domains with distinct identities and provide positional cues for the subsequent formation of specialized structures. A well‐defined example of such spatial patterning is the maize leaf, which is subdivided along the proximal‐distal axis into a distal blade and a proximal sheath, with the ligular region forming at their junction (Fowler & Freeling, 1996; Johnston et al., 2014; Sylvester et al., 1990). The ligular region gives rise to two specialized structures, the thin membranous ligule and the wedge‐like auricles, through a highly ordered developmental sequence involving blade‐sheath boundary establishment, preligule band (PLB) formation, and subsequent ligule/auricle differentiation (Sylvester et al., 1990). Of these developmental events, ligule development from the adaxial epidermis provides a particularly attractive system for investigating how positional information is translated into localized cell‐fate specification and tissue outgrowth, because its initiation is spatially restricted and also morphologically recognizable. Furthermore, since several regulators required for ligule development have been found also to function in the initiation or patterning of other lateral organs, including leaf primordia and tassels (Bertolini et al., 2025; Johnston et al., 2014; Lewis et al., 2014; Li et al., 2026; Toriba et al., 2019; Walsh & Freeling, 1999), this suggests that related developmental programs can operate across distinct morphogenetic contexts. However, the molecular mechanisms that determine how ligule cell fate is specified remain poorly understood.

Genetic analyses and molecular studies that have established an initial framework for ligule development in maize and other grasses started with classical mutant studies that identified *LIGULELESS1/2* (*LG1/2*) as core regulators of ligule formation (Becraft et al., 1990; Lee et al., 2007; Liu, Cao, et al., 2019; Moreno et al., 1997; Walsh & Freeling, 1999; Wang et al., 2021). Very recently, ZmLG1 has been shown to directly upregulate the expression of the auxin transporter genes *PIN‐FORMED1a/b/c* (*ZmPIN1a/b/c*) in the preligule region (Zhong et al., 2025), linking transcriptional regulation to local auxin transport during ligule development. Genetic evidence has also established roles for members of the *WUSCHEL‐LIKE HOMEOBOX* (*WOX*) and *BLADE‐ON‐PETIOLE* (*BOP*) families in the development of the ligule (Dai et al., 2007; Satterlee et al., 2023; Tavakol et al., 2015; Toriba et al., 2019). Transcriptomic and spatial expression studies have further identified genes enriched in the PLB or developing ligule, including *ZmLG3*, *SQUAMOSA PROMOTER BINDING PROTEIN3* (*ZmSBP3*), *BEL1‐like homeodomain 12/14* (*ZmBEL12/14*), *CUP‐SHAPED COTYLEDON* (*CUC*) genes, *VESTIGIAL GLUME1* (*ZmVG1*), and *INDETERMINATE DOMAIN31* (*ZmIDD31*) (Evans et al., 2026; Johnston et al., 2014; Li et al., 2026). However, the transcriptional dynamics underlying ligule initiation and subsequent outgrowth remain poorly resolved.

Precise elucidation of ligule developmental mechanisms requires profiling cell‐fate specification at the highest possible spatiotemporal resolution. Ligule development can be broadly divided into PLB formation and fringe outgrowth, each comprising morphologically defined substages (Neher et al., 2023). Laser microdissection RNA sequencing (LM‐RNA‐seq) analysis of the PLB has identified hundreds of ligule‐upregulated genes and revealed extensive activation of transcription factor families (e.g., *Homeobox*, *MADS‐box*, *AP2/EREBP*, *SBP*, and *MYB*) and phytohormone‐related pathways, particularly those associated with auxin transport and response (Johnston et al., 2014). However, because these studies profiled microdissected tissues rather than individual cells, they could not resolve the cellular heterogeneity of the developing ligular region or define cell‐type‐specific transcriptional transitions. More recently, cell‐resolved transcriptomic studies have examined the maize ligular region after PLB formation. Wang et al. (2024) established a single‐nucleus transcriptomic atlas of the fully formed ligular region, resolving its cellular heterogeneity and specialized tissues. Evans et al. (2026) identified ligule‐associated cell populations from Early‐Fringe to Late‐Mid‐Fringe, with a primary focus on the acquisition of auricle cell fate. However, neither study included the PLB stage, leaving the cell‐resolved transcriptional progression from PLB formation to subsequent ligule outgrowth incompletely understood.

Here, we used snRNA‐seq to profile maize ligular‐region tissues at the PLB formation and ligule outgrowth stages, revealing a distinctive ligule population supported by multiple lines of evidence and reconstructed an inferred developmental trajectory. To dissect the underlying regulatory mechanisms, we constructed gene co‐expression networks at single‐nucleus resolution to uncover core transcriptional programs associated with ligule development. Guided by these networks, we identified ZmIDD30, a transcription factor specifically expressed in the developing ligule, as a candidate regulator of ligule development. We further demonstrated that ZmIDD30 directly binds to the promoters of *ZmVG1* and *ZmPIN1c*, and thereby activates their transcription. Collectively, this study provides a single‐nucleus transcriptional atlas of maize ligule development, identifies regulatory components associated with this process, and offers a valuable resource for investigating the molecular basis of ligule morphogenesis.

## RESULTS

### Single‐nucleus transcriptomic profiling reveals cellular heterogeneity in the maize ligular region from PLB formation to ligule outgrowth

To capture the PLB formation and subsequent ligule outgrowth for single‐nucleus profiling, we first surveyed the ligular regions of successive leaves from 12‐day‐old maize seedlings using cryo‐scanning electron microscopy (cryo‐SEM) (Figure 1a). At the adaxial base of the fourth leaf, the blade‐sheath boundary comprised a narrow band of densely packed, short rectangular cells, characteristic of a previously described mid‐to‐late PLB stage (Neher et al., 2023). By contrast, the adaxial base of the third leaf displayed a protruding ligule fringe composed of elongated cells with an uneven, corrugated margin, characteristic of the mid‐fringe outgrowth stage (Neher et al., 2023). Based on these morphological features, we designated the fourth‐leaf ligular region as the PLB formation stage (S1) and the third‐leaf ligular region as the ligule outgrowth stage (S2) and collected the corresponding tissues for snRNA‐seq.

**Figure 1:**
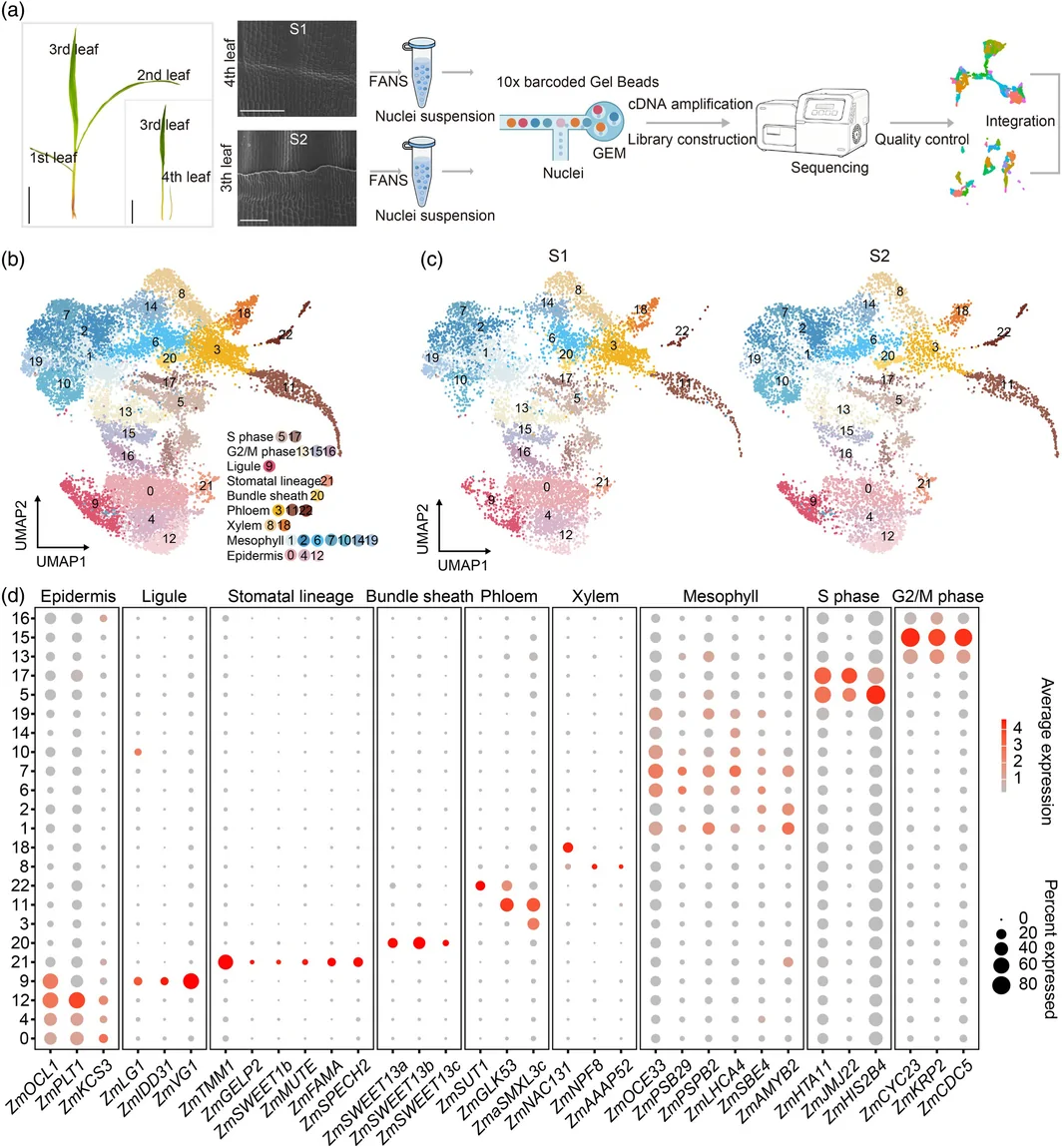
Single‐nucleus transcriptomic landscape of the developing maize ligular region. (a) Experimental workflow for snRNA‐seq of the maize ligular region. S1 and S2 were collected from the fourth and third leaves of 12‐day‐old seedlings, respectively, corresponding to the preligule band stage and the ligule outgrowth stage. Scale bars: black, 5 cm; white, 100 μm. (b) UMAP visualization of the integrated snRNA‐seq dataset from S1 and S2. Each dot represents a single nucleus, and colors indicate 23 transcriptionally distinct clusters. (c) Split UMAP plots showing nuclei from S1 and S2. (d) Dot plot showing representative marker genes used for cell‐type annotation across clusters. Dot sizes represent the percentage of nuclei expressing each gene, and dot color indicates the average expression level.

Nuclei from both samples were isolated by fluorescence‐activated nuclei sorting (FANS) (Galbraith et al., 1983; Sun et al., 2022), which greatly reduced contamination by debris and specifically enriched intact nuclei with clear 2C/4C DNA‐content peaks (Figure S1). snRNA‐seq libraries were then generated from these sorted nuclei and sequenced. Post‐sequencing quality control was applied to the gene‐barcode matrix by evaluating UMI counts, detected gene numbers, and predicted doublets, followed by the removal of low‐quality nuclei and putative doublets (Figure S2a–f). The retained nuclei showed low proportions of both mitochondrial and chloroplast transcripts, indicating limited organellar transcript contamination (Figure S2g, h). Following filtering, 15 711 high‐quality nuclei were retained, including 8757 from S1 and 6954 from S2. The median numbers of detected genes per nucleus were 1837 and 2162 in S1 and S2, respectively, with corresponding median UMI counts of 2372 and 2997 (Table S1). We then used the matched S1 and S2 snRNA‐seq datasets, generated within the same experimental framework, for downstream clustering and trajectory analyses to minimize potential technical and developmental‐stage effects arising from cross‐study integration. Unsupervised clustering of the integrated datasets identified 23 transcriptionally distinct clusters (Figure 1b). All major clusters were represented in both samples, although their relative abundances differed between stages (Figure 1c, Figure S3).

We next assigned cell identities to these clusters based on previously reported marker genes (Table S2). Epidermal marker genes, including *OUTER CELL LAYER1* (*ZmOCL1*) (Ingram et al., 1999), *PHOSPHOLIPID TRANSFER PROTEIN HOMOLOG1* (*ZmPLT1*) (Takacs et al., 2012), and *3‐KETOACYL‐COA SYNTHASE3* (*ZmKCS3*) (Wang et al., 2024), were preferentially expressed in clusters 0, 4, 9, 12, and 21 (Figure 1d; Figure S4a). Across these epidermal clusters, genes associated with guard cell and subsidiary cell development, including *TOO MANY MOUTHS‐LIKE1* (*ZmTMM1*, a maize homolog of Arabidopsis *TMM*) (Bhave et al., 2008), *SPEECHLESS2* (*ZmSPCH2*) (Raissig et al., 2016), *ZmMUTE* (Hake et al., 2019), *ZmFAMA* (Wu et al., 2019), *GDSL ESTERASE/LIPASE2* (*ZmGELP2*) (Sun et al., 2022), and *ZmSWEET1b* (Wang et al., 2019), were specifically expressed in cluster 21 (Figure 1d, Figure S4b, c), identifying this cluster as the stomatal lineage.

We next characterized vascular‐related clusters using maize homologs of known Arabidopsis vascular marker genes. Phloem‐associated marker genes, such as *SUCROSE TRANSPORTER1* (*ZmSUT1*) (Bezrutczyk et al., 2021), *G2‐LIKE transcription factor 53* (*ZmGLK53*, a maize homolog of Arabidopsis *ALTERED PHLOEM DEVELOPMENT*) (Bonke et al., 2003), and *SMAX1‐LIKE3c* (*ZmaSMXL3c*, a maize homolog of Arabidopsis *SMXL3*) (Wallner et al., 2017) were preferentially expressed in clusters 3, 11, and 22 (Figure 1d; Figure S4d), indicating that these clusters correspond to the phloem. Xylem‐associated marker genes, including *ZmNAC131* (a maize homolog of Arabidopsis *VASCULAR‐RELATED NAC‐DOMAIN6*) (Kubo et al., 2005), *NRT1/PTR FAMILY8* (*ZmNPF8*, a maize homolog of Arabidopsis *NPF6.2*) (Kim et al., 2021), and *AMINO ACID PERMEASE52* (*ZmAAAP52*, a maize homolog of Arabidopsis *AAP6*) (Okumoto et al., 2002), showed markedly enriched expression in clusters 8 and 18 (Figure 1d, Figure S4e), defining the xylem population. Additionally, transcripts for *ZmSWEET13a/b/c* (Bezrutczyk et al., 2021), three members of the *SWEET* sucrose transporter family previously reported as bundle sheath markers, accumulated exclusively in cluster 20 (Figure 1d, Figure S4f), identifying this cluster as the bundle sheath population.

Established marker genes of mesophyll cells involved in photosynthesis, including *OXYGEN EVOLVING COMPLEX33* (*ZmOEC33*), *PHOTOSYSTEM II SUBUNIT29* (*ZmPSB29*), *PHOTOSYSTEM II OXYGEN EVOLVING POLYPEPTIDE2* (*ZmPSPB2*) (Bezrutczyk et al., 2021), *LIGHT HARVESTING COMPLEX A4* (*ZmLHCA4*), *STARCH BRANCHING ENZYME4* (*ZmSBE4*), and *BETA AMYLASE2* (*ZmAMYB2*) (Sun et al., 2022), were predominantly expressed in clusters 1, 2, 6, 7, 10, 14, and 19 (Figure 1d, Figure S4g).

We also identified proliferating cell populations based on the expression of cell‐cycle‐related genes. The expression patterns of *ZmHTA11* (a maize homolog of Arabidopsis *HISTONE H2A11*), *JUMONJI transcription factor 22* (*ZmJMJ22*), and *HISTONE 2B4* (*ZmHIS2B4*) (Satterlee et al., 2020) indicated that S‐phase cells were over‐represented within clusters 5 and 17 (Figure 1d, Figure S4h). Meanwhile, the enriched expression of *KINESIN‐RELATED PROTEIN2* (*ZmKRP2*), *CYCLIN23* (*ZmCYC23*), and *CELL DIVISION CONTROL PROTEIN HOMOLOG5* (*ZmCDC5*) (Satterlee et al., 2020; Yuan et al., 2024) revealed that clusters 13, 15, and 16 corresponded to cells in G2/M (Figure 1d, Figure S4i).

Together, this marker‐based annotation defines the major cell populations in the maize ligular region during the PLB formation and ligule outgrowth stages and provides a basis for identifying cell populations associated with ligule development.

### Transcriptomic comparison and spatial validation identify a ligule population

For the identification of the ligule population, we first examined the expression of previously reported maize ligule marker genes across the single‐nucleus landscape (Table S2, Figure S5a). Among these marker genes, several known ligule‐associated genes, particularly *ZmLG1* (Johnston et al., 2014), *ZmIDD31* and *VESTIGIAL GLUME1* (*ZmVG1*) (Evans et al., 2026), were preferentially expressed in cluster 9 of the epidermal lineages (Figure 1d, Figure S5b), providing initial evidence that cluster 9 represents an epidermis‐derived ligule population.

To further support the ligule identity of cluster 9, we leveraged previously published LM‐RNA‐seq data for comparative analysis. In the published dataset, 314 and 235 ligule‐upregulated genes were identified from all‐cell‐layer and adaxial epidermal samples, respectively (Figure 2a) (Johnston et al., 2014). We intersected these two LM‐RNA‐seq‐derived gene sets with the differentially expressed genes (DEGs) across all clusters in our snRNA‐seq dataset (Tables S3 and S4). Hypergeometric tests revealed that the ligule‐upregulated genes from both all‐cell‐layer and adaxial epidermal samples were significantly enriched in cluster 9, with 71 (*P* = 0.002) and 83 overlapping genes (*P* = 1.3 × 10^−13^), respectively (Figure 2b–d). The all‐cell‐layer ligule gene set was additionally enriched in mesophyll cluster 10. Previous histological analyses have shown that mesophyll cells underlying the developing ligule undergo periclinal divisions and contribute to localized tissue thickening during ligule initiation (Sylvester et al., 1990). Thus, the enrichment in cluster 10 may reflect a ligular‐region‐associated developmental program in a subset of mesophyll cells. Nevertheless, the consistent and stronger enrichment of both LM‐RNA‐seq‐derived gene sets in cluster 9 provides independent support for its annotation as the ligule population.

**Figure 2:**
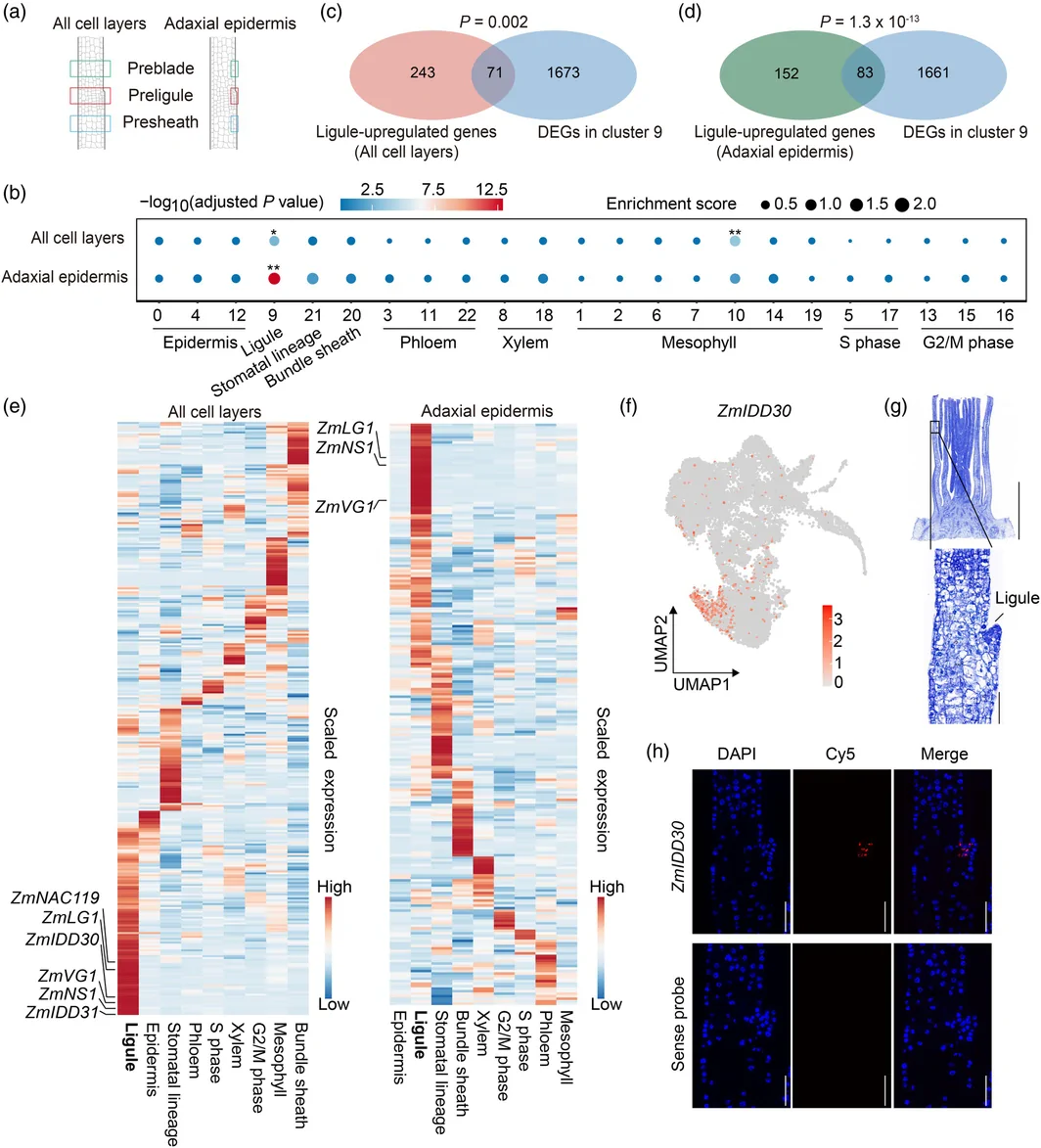
Comparative analysis of LM‐RNA‐seq and snRNA‐seq datasets and validation of ligule identity by RNA FISH of *ZmIDD30*. (a) Schematic diagram illustrating the sampling strategy of the published laser microdissection RNA sequencing (LM‐RNA‐seq) dataset (Johnston et al., 2014). (b) Dot plot showing the enrichment of LM‐RNA‐seq‐derived ligule‐upregulated genes from all‐cell‐layer and adaxial epidermis samples among DEGs from each snRNA‐seq cluster. Dot color denotes the ‐log10 (adjusted *P* value) from hypergeometric tests, and dot size denotes the enrichment score. Asterisks indicate adjusted *P* value thresholds (*adjusted *P* < 0.01, **adjusted *P* < 0.001). (c, d) Venn diagrams showing the overlap between DEGs in snRNA‐seq cluster 9 and ligule‐upregulated genes from the LM‐RNA‐seq dataset in all‐cell‐layer samples (c) and adaxial epidermis samples (d). (e) Heatmaps showing the expression patterns of LM‐RNA‐seq‐derived ligule‐upregulated genes across cell types identified in the snRNA‐seq dataset. Genes previously reported to be involved in ligule development and candidate ligule‐associated genes are indicated. (f) Feature plot showing the expression distribution of *ZmIDD30* across all snRNA‐seq clusters. (g) Longitudinal section of a 10‐day‐old maize shoot apex after toluidine blue O (TBO) staining. The enlarged region contains an emerging ligule whose morphology is comparable to the S2 ligule outgrowth stage defined in Figure 1. The FISH material was collected independently from the samples used for snRNA‐seq. Scale bars: 500 μm (top) and 50 μm (bottom). (h) RNA fluorescence *in situ* hybridization (FISH) of *ZmIDD30* in the ligular region using a Cy5‐labeled *ZmIDD30* probe (red signal); nuclei were counterstained with DAPI (blue signal). The upper row shows hybridization with the antisense *ZmIDD30* probe, and the lower row shows the corresponding sense‐probe control. Scale bars: 50 μm.

We subsequently examined the expression patterns of the two sets of ligule‐upregulated genes from LM‐RNA‐seq across the snRNA‐seq atlas. Most of these genes exhibited high expression levels in the putative ligule population (cluster 9), including known ligule‐associated genes such as *ZmLG1*, *ZmNAC119* (Johnston et al., 2014), *NARROW SHEATH1* (*ZmNS1*) (Satterlee et al., 2023), and *ZmVG1* (Evans et al., 2026; Liu, Zhao, et al., 2019) (Figure 2e). Notably, our reanalysis of the published LM‐RNA‐seq dataset showed that *ZmIDD30*, like the previously reported ligule‐enriched gene *ZmIDD31* (Evans et al., 2026), preferentially accumulated in ligule samples. Both genes were also specifically enriched in cluster 9 in our snRNA‐seq dataset (Figure 2f, Figures S5b and S6). Because direct spatial evidence for *ZmIDD30* expression in the developing ligule was lacking, we selected it for fluorescence *in situ* hybridization (FISH) analysis. Consistent with the transcriptomic results, *ZmIDD30* transcripts were specifically localized to the developing ligule (Figure 2g, h), providing direct spatial evidence supporting the ligule identity of cluster 9.

### Trajectory analysis reveals sequential transcriptional programs during ligule development

Given that the ligule originates from the epidermis, we first reconstructed a pseudotime trajectory using epidermal cell populations (Figure 3a). The resulting trajectory began at State I, progressed along a shared epidermal path to State II, and then bifurcated into two branches (Figure 3b). Branch 1 extended toward terminus III and was dominated by the ligule‐associated cluster 9. Consistently, ligule‐specific marker genes, including *ZmLG1, ZmIDD31, and ZmVG1*, were specifically enriched along this branch (Figure S7a), supporting its identification as the ligule differentiation lineage. Cells along Branch 1 showed a progressive shift in sample origin: cells in the early portion were predominantly derived from S1, whereas those toward terminus III were mainly derived from S2, consistent with developmental progression from the PLB formation stage to ligule outgrowth (Figure 3b). Branch 2, extending from State II to terminus IV, contained clusters 12 and 21 (Figure 3b). Stomatal‐lineage marker genes were enriched toward the terminal region of this branch, consistent with the assignment of cluster 21 as the stomatal lineage (Figure 3b; Figure S7b).

**Figure 3:**
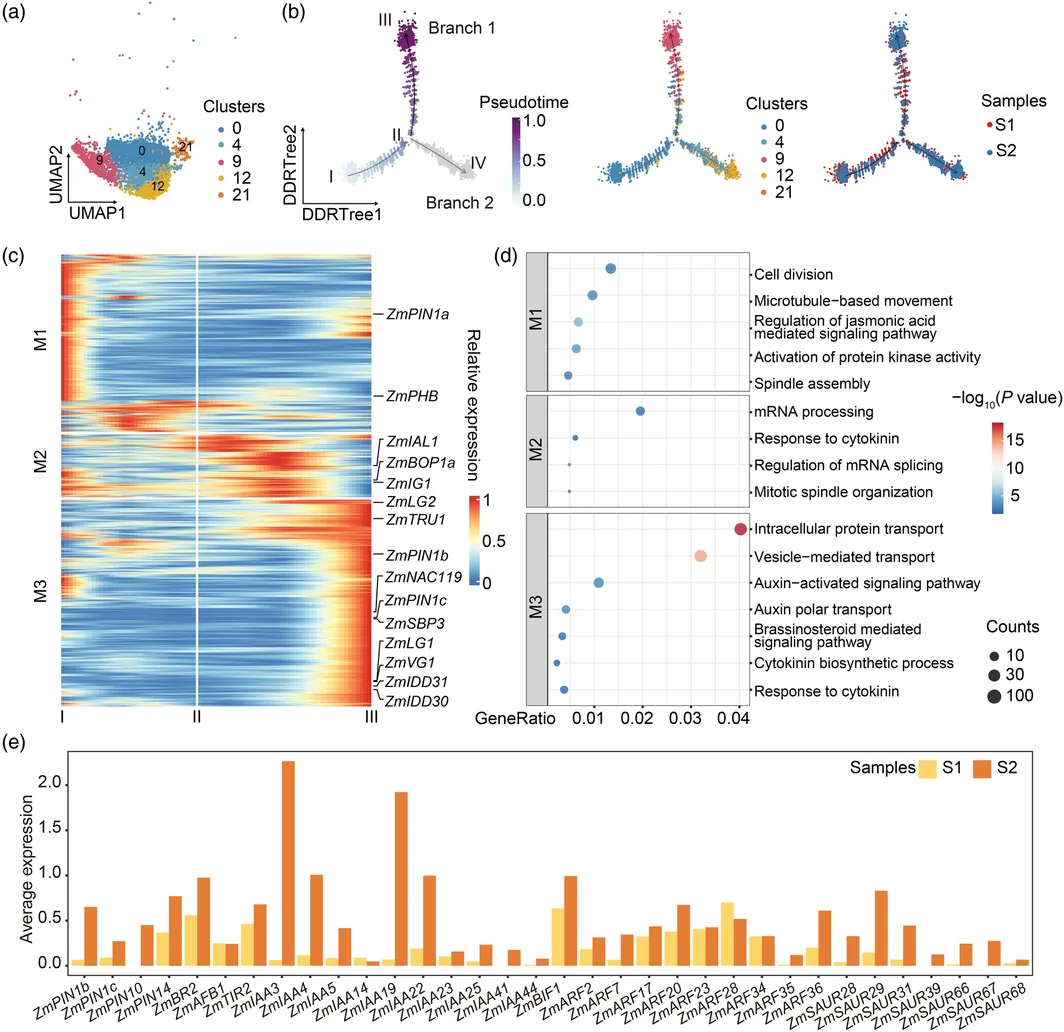
Developmental trajectory of maize ligule differentiation within the epidermal lineage. (a) UMAP visualization of the epidermal lineage subsetted from the integrated snRNA‐seq dataset for trajectory reconstruction. (b) Pseudotime trajectory of the epidermal lineage. Panels show, from left to right, nuclei colored by pseudotime, cluster identity, and sample origin. (c) Transcriptional dynamics along the ligule differentiation trajectory. The heatmap displays genes partitioned into three modules based on their expression dynamics along the I‐II‐III trajectory. (d) Dot plot showing representative Gene Ontology (GO) terms enriched in the three dynamic expression modules shown in (c). Dot color represents ‐log_10_(*P* value), and dot size represents the number of genes associated with each term. (e) Bar plot showing log‐transformed average expression levels of genes involved in auxin polar transport and signaling pathway in the ligule cluster from S1 and S2 samples.

To further elucidate the sequential transcriptional programs accompanying the progression from the epidermis to the ligule, we performed hierarchical clustering of genes expressed along the I‐II‐III axis, which identified three distinct dynamic expression modules. Module 1 (M1) genes exhibited higher expression levels prior to trajectory bifurcation (I‐II) and subsequently declined (Figure 3c). Module 2 (M2) genes showed a transient activation pattern, peaking shortly after branch point II and then gradually decreasing. This early‐response module harbored previously reported boundary formation regulators, including *INDETERMINATE GAMETOPHYTE1* (*ZmIG1*), its paralog *IG1‐AS2 LIKE1* (*ZmIAL1*) (Evans, 2007), and the *BOP‐like* gene *ZmBOP1a* (Evans et al., 2026) (Figure 3c). Module 3 (M3) genes increased progressively during ligule development and included known ligule‐associated genes such as *ZmLG1*, *ZmNAC119*, *ZmSBP3*, and *ZmVG1*, as well as the auxin efflux genes *ZmPIN1b* and *ZmPIN1c* (Zhong et al., 2025). Notably, *ZmIDD30* and *ZmIDD31* were co‐expressed within M3, pointing to their potential involvement in ligule development (Figure 3c).

Functional enrichment analysis revealed that M1 was significantly enriched for Gene Ontology (GO) terms associated with cell proliferation and structural reorganization, including cell division, spindle assembly, microtubule‐based movement, the activation of protein kinase activity, and the regulation of jasmonic acid‐mediated signaling (Figure 3d, Table S5). This transcriptional profile likely reflects an actively proliferating epidermal state before divergence toward the ligule trajectory. Immediately following the bifurcation, M2 genes were enriched for RNA processing, mitotic spindle organization, and responses to cytokinin (Figure 3d, Table S5), consistent with the active cell divisions during the PLB formation stage (Neher et al., 2023). M3 genes showed enrichment for intracellular and vesicle‐mediated protein transport, brassinosteroid‐mediated signaling, cytokinin biosynthesis and response, polar auxin transport, and auxin signaling pathways (Figure 3d, Table S5). Consistent with the functional enrichment of M3, genes associated with these hormone‐related pathways showed higher average expression in S2 than in S1 (Figure 3e and Figure S8). Together, these patterns revealed a progressive shift from cell‐division‐associated programs to hormone‐responsive and intracellular transport processes along the ligule trajectory.

### Co‐expression network analysis reveals stage‐biased modules and prioritizes candidate regulators of ligule development

To investigate potential transcriptional regulatory relationships during ligule development, we constructed gene co‐expression networks using the singleCellGGM algorithm (Xu et al., 2024). After filtering out genes expressed in fewer than 10 cells, a total of 59 466 significant co‐expression gene pairs (FDR <0.05) were identified and used to construct the network. Application of the Markov cluster algorithm subsequently partitioned the network into 87 co‐expression modules, with each module containing at least 10 genes. Among these singleCellGGM‐derived co‐expression modules, module 06 (M06, 267 genes, Table S6) and module 27 (M27, 69 genes, Table S6) were specifically enriched in the ligule. These two modules displayed stage‐biased expression patterns, with M27 enriched in S1 and M06 enriched in S2 (Figure 4a).

**Figure 4:**
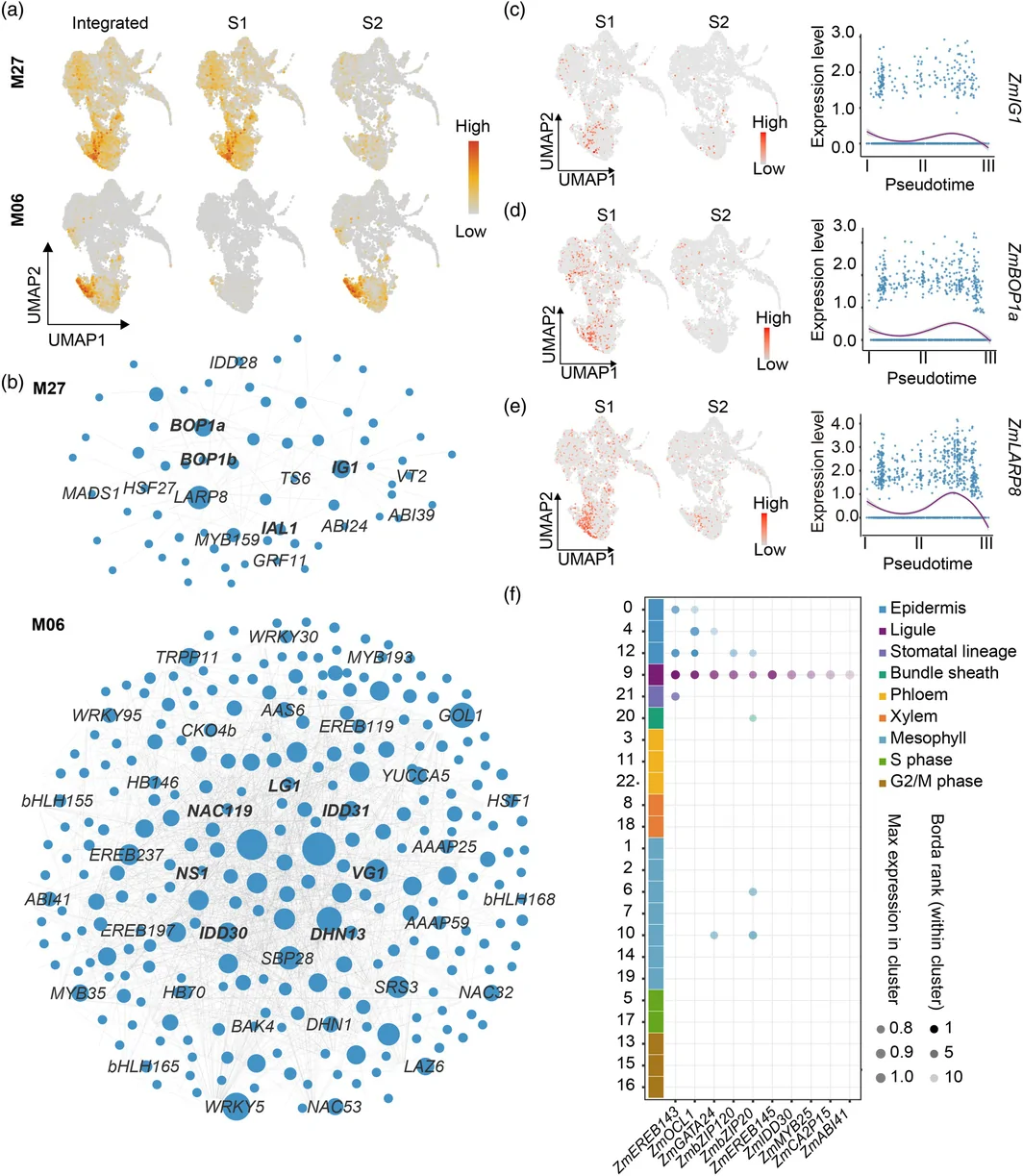
Co‐expression network analysis identifies candidate regulators of maize ligule development. (a) UMAP visualization of expression scores for the singleCellGGM‐derived co‐expression modules M27 and M06 in the integrated, S1, and S2 datasets. (b) Co‐expression networks of M27 and M06. Node size reflects network connectivity, and labeled nodes indicate representative genes. (c–e) Feature plots showing the expression of representative M27 genes in S1 and S2, together with their expression dynamics along the epidermis‐to‐ligule pseudotime trajectory: *ZmIG1* (c), *ZmBOP1a* (d), and *ZmLARP8* (e). Dots in the pseudotime plots represent individual nuclei, and solid lines indicate LOESS‐fitted expression trends. (f) Dot plot showing the top‐ranked candidate transcription factors identified by MINI‐EX for the ligule cluster. The colored bar indicates the cell‐type annotation of each cluster. Dot grayscale represents the Borda rank within each cluster, with darker dots indicating higher ranks, and dot size represents the maximum expression level within the cluster.

M27 contained several boundary‐associated genes, including *ZmIG1*, *ZmIAL1*, *ZmBOP1a*, and *ZmBOP1b* (Figure 4b). Except for *ZmBOP1b*, which exhibited relatively low expression, *ZmIG1*, *ZmIAL1*, and *ZmBOP1a* displayed elevated expression levels during S1 (Figure 4c, d, Figure S9a, b). Along the pseudotime trajectory (II–III), these genes consistently showed early activation followed by progressive decline (Figure 4c, d, Figure S9a, b), suggesting that M27 is primarily associated with the PLB formation stage. In addition, *ZmLARP8*, encoding a La‐related protein, and *ZmIDD28*, both assigned to this module, represented additional candidate genes potentially involved in ligule initiation (Figure 4e, Figure S9c).

In contrast, the S2‐enriched M06 contained established ligule‐associated regulators, such as *ZmLG1*, *ZmVG1*, and *ZmNS1*, alongside genes previously reported to accumulate in the ligule, including *ZmNAC119*, *ZmDHN13* (Leiboff et al., 2021), and *ZmIDD31* (Figure 4b). M06 also contained genes from multiple transcription factor families, including *AP2/ERF*, *bHLH*, *WRKY*, *NAC*, and *MYB*, suggesting that this module captures a broad transcriptional program associated with ligule outgrowth. M06 also contained phytohormone‐related genes associated with auxin biosynthesis (*ZmYUCCA5*), cytokinin metabolism (*ZmCKO4b*), and brassinosteroid signaling (*ZmBAK4*), suggesting that multiple hormone pathways may coordinately contribute to ligule development.

To further identify candidate regulators associated with this process, we employed the MINI‐EX pipeline to prioritize transcription factors enriched in the ligule cluster. *ZmIDD30* and *ZmABI41* ranked among the top 10 candidates and were both assigned to module M06 (Figure 4b, f). *ZmIDD30* encoded a previously uncharacterized C2H2 zinc finger transcription factor. Stage‐resolved feature plots showed that *ZmIDD30* transcripts were already detected in S1 at the PLB formation stage and became more prominent in S2 during ligule outgrowth (Figure S9d). Given its ligule‐enriched expression confirmed by FISH and its sustained expression across PLB formation and ligule outgrowth, *ZmIDD30* was selected for further functional characterization.

### ZmIDD30 directly binds to the promoters of *ZmVG1* and *ZmPIN1c* and activates their transcription

Subcellular localization analysis in maize protoplasts showed that ZmIDD30 was exclusively localized to the nucleus (Figure 5a), consistent with a potential role in transcriptional regulation. To prioritize candidate downstream genes potentially associated with ZmIDD30 function, we examined its pseudotime expression pattern along the ligule trajectory together with representative ligule‐associated genes. *ZmVG1*, *ZmLG1*, *ZmPIN1b*, and *ZmPIN1c* showed a progressive transcriptional accumulation pattern similar to that of *ZmIDD30* (Figures 3c, 5b, Figure S9d–g).

**Figure 5:**
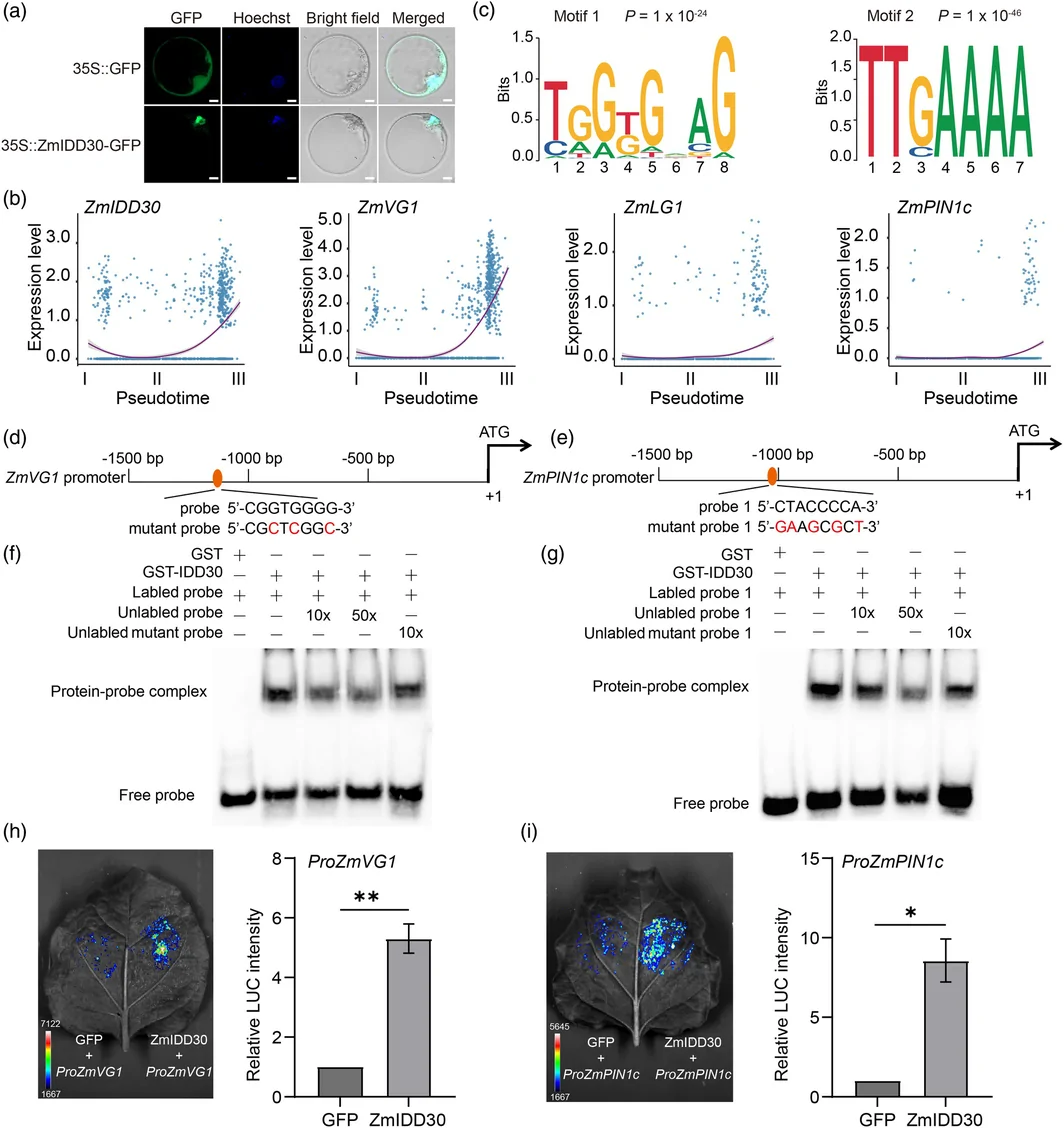
ZmIDD30 directly binds to the promoters of *ZmVG1* and *ZmPIN1c* and activates their expression. (a) Subcellular localization of ZmIDD30‐GFP in maize protoplasts. The 35S::GFP construct was used as a control. Scale bars: 10 μm. (b) Pseudotime expression patterns of *ZmIDD30*, *ZmVG1*, *ZmLG1*, and *ZmPIN1c* along the epidermis‐ligule differentiation trajectory. Dots represent individual nuclei, and lines represent LOESS‐fitted expression trends. (c) Enriched sequence motifs identified from ZmIDD30‐associated CUT&Tag peaks. Motif 1 and Motif 2 correspond to [T/C]GG[T/G]GNNG and TT[G/C]AAAA, respectively. (d, e) Motif 1 sites identified within approximately 1.5 kb of the promoter regions of *ZmVG1* (d) and *ZmPIN1c* (e). The Motif 1 site in the *ZmPIN1c* promoter is located on the antisense strand. (f, g) EMSA assays showing the binding of ZmIDD30 to Motif 1 elements in the *ZmVG1* promoter (f) and *ZmPIN1c* promoter (g). Binding specificity was examined using 10× and 50× unlabeled competitor probes and 10× mutant competitor probes, with GST protein used as a negative control. (h, i) Luciferase reporter assays showing that ZmIDD30 activates the expression of *ZmVG1* (h) and *ZmPIN1c* (i) in *Nicotiana benthamiana* leaves. Relative LUC intensity is shown as mean ± SE (*n* = 3). Statistical significance was assessed by Student's *t*‐test; asterisks indicate significant differences (*P < 0.05, **P < 0.01).

To investigate the DNA‐binding motifs of ZmIDD30, we first performed CUT&Tag assays. The CUT&Tag data showed high reproducibility among the three technical replicates, with strand cross‐correlation analysis further supporting the overall quality (Figure S10). Motif enrichment analysis identified two top‐ranked motifs: [T/C]GG[T/G]GNNG (designated as Motif 1) and TT[G/C]AAAA (designated as Motif 2) (Figure 5c, Table S7), both of which were matched by HOMER to motifs associated with C2H2 zinc finger transcription factors. Motif 2 contained an A‐rich [G/C]AAAA element resembling the DAP‐seq‐defined binding motif of Arabidopsis IDD4, which contains a GACAAAA sequence, and was broadly consistent with the previously established DNA‐binding preferences of IDD family proteins (Kozaki et al., 2004; O'malley et al., 2016; Völz et al., 2019). Because CUT&Tag was performed in transiently transformed leaf protoplasts derived from etiolated seedlings rather than developing ligule, ligule‐context‐dependent binding events may not have been fully captured. We subsequently conducted a targeted *cis*‐element scan within the 3 kb upstream promoter regions of the co‐expressed candidate genes. Sequence analysis revealed that the promoters of *ZmVG1*, *ZmLG1*, and *ZmPIN1c* harbored Motif 1 elements, whereas the *ZmPIN1b* promoter did not. Furthermore, the *ZmPIN1c* promoter uniquely contained an additional Motif 2 element (Figure 5d, e, Figure S11a, b). These sequence features suggested that these genes were potential downstream targets of ZmIDD30.

To test whether ZmIDD30 directly binds to these candidate sites *in vitro*, we purified the recombinant ZmIDD30 protein and performed electrophoretic mobility shift assays (EMSAs) (Figure S12a). ZmIDD30 specifically bound to the Motif 1 elements within the *ZmVG1* and *ZmPIN1c* promoters (Figure 5f, g). In contrast, ZmIDD30 showed only non‐specific binding to the Motif 1 element in the *ZmLG1* promoter and did not specifically interact with the Motif 2 element in the *ZmPIN1c* promoter (Figure S11a, b). Subsequently, transient luciferase reporter assays in *Nicotiana benthamiana* leaves demonstrated that ZmIDD30 significantly activated the transcription of *ZmVG1* and *ZmPIN1c* (Figure 5h, i). Together, these results support *ZmVG1* and *ZmPIN1c* as direct transcriptional targets of ZmIDD30.

We further investigated ZmIDD31, a closely related paralog that formed a sister pair with ZmIDD30 in the phylogenetic analysis (Liu et al., 2024) and has also been reported to be expressed during ligule outgrowth (Evans et al., 2026). Similar to ZmIDD30, ZmIDD31 was exclusively localized to the nucleus in protoplasts, supporting its potential function as a transcription factor (Figure S13a). Although *ZmIDD31* showed a pseudotime expression pattern similar to those of *ZmIDD30*, *ZmVG1*, *ZmPIN1c*, and *ZmLG1* along the ligule developmental trajectory (Figure S13b), it did not show detectable binding in EMSAs to the motif‐containing promoter fragments from *ZmVG1*, *ZmPIN1c*, or *ZmLG1* (Figures S12b and S13c–e). These results suggest that ZmIDD30 and ZmIDD31 may have diverged in their DNA‐binding properties during ligule development.

## DISCUSSION

Ligule development in maize provides a classical model for studying how positional information at the blade‐sheath boundary is translated into localized cell‐fate specification and subsequent tissue outgrowth (Johnston et al., 2014). Although previous genetic and transcriptomic studies have identified several key ligule‐associated regulators, including *LG1*, *LG2*, and members of the *BOP* and *WOX* gene families (Becraft et al., 1990; Lee et al., 2007; Moreno et al., 1997; Satterlee et al., 2023; Toriba et al., 2019; Walsh et al., 1998; Wang et al., 2021), the cellular progression of ligule development from the PLB formation stage to the ligule outgrowth stage has remained unclear. In this study, we generated a single‐nucleus atlas of the maize ligular region across the PLB formation and ligule outgrowth stages. This atlas enabled us to identify a ligule population within the heterogeneous ligular region, reconstruct its developmental trajectory, and integrate co‐expression analysis with molecular validation to uncover regulatory components underlying ligule development.

A prerequisite for reconstructing this developmental progression is the reliable identification of ligule‐associated cells within the single‐nucleus dataset. Previous anatomical studies have shown that the maize ligule arises from the adaxial epidermis, and transcriptomic studies have identified multiple genes expressed in the PLB and developing ligule (Johnston et al., 2014; Sylvester et al., 1990). The spatial expression of key ligule‐associated genes has been validated by RNA *in situ* hybridization (Evans et al., 2026; Johnston et al., 2014; Leiboff et al., 2021; Satterlee et al., 2023), providing reliable markers for identifying ligule‐associated cells. Building on this foundation, we integrated these genes and LM‐RNA‐seq signatures into a single‐nucleus framework. Cluster 9 was annotated as a ligule population because it was enriched for known ligule‐related genes, including *ZmLG1*, *ZmVG1*, and *ZmIDD31*, and also showed strong overlap with ligule‐upregulated genes from previous LM‐RNA‐seq datasets. This assignment was further supported by FISH showing the accumulation of *ZmIDD30* transcripts in the developing ligule. Identification of this ligule population enabled us to trace transcriptional changes from PLB formation to ligule outgrowth and provided the basis for subsequent trajectory and regulatory network analyses.

Trajectory and co‐expression analyses converged on an early‐to‐late transcriptional transition during ligule development. The transient pseudotime module M2 and the S1‐enriched co‐expression module M27 contained boundary‐associated genes, including *ZmIG1*, *ZmIAL1*, and *ZmBOP1a*. By contrast, the progressively activated pseudotime module M3 and the S2‐enriched co‐expression module M06 contained established and candidate ligule‐associated genes, including *ZmLG1*, *ZmNS1*, *ZmNAC119*, *ZmDHN13*, *ZmVG1*, *ZmIDD31*, and *ZmIDD30*. Together, these complementary analyses support a transcriptional progression from a PLB‐associated boundary program toward a program associated with ligule outgrowth.

This temporal organization is broadly consistent with previous network analyses of maize leaf primordia (Leiboff et al., 2021). The expression of genes involved in inflorescence and floral development, including *TASSEL SEED6* (*ZmTS6*), *VANISHING TASSEL2* (*ZmVT2*), and *ZmMADS1*, together with the roles of *ZmLG1* and *ZmVG1* in both ligule and tassel development (Bertolini et al., 2025; Chuck et al., 2007; Heuer et al., 2001; Lewis et al., 2014; Liu, Zhao, et al., 2019; Phillips et al., 2011), suggests that ligule and inflorescence development share regulatory components. Such shared components may reflect the conservation and rewiring of ligule and tassel branch regulatory networks described by Bertolini et al. (2025). Together, our single‐nucleus analysis extends this concept in which gene expression programs associated with boundary formation and lateral organ development are redeployed during ligule initiation and outgrowth (Johnston et al., 2014).

Within the progressively activated program, ZmIDD30 emerged as a candidate regulator of ligule development. Although recent cell‐resolved work identified ZmIDD31 as a ligule marker during fringe development (Evans et al., 2026), our stage‐resolved data show that its closely related paralog ZmIDD30 is already expressed during PLB formation and becomes more prominent during subsequent ligule outgrowth. The two proteins differed in their recognition of the promoter fragments examined in this study. ZmIDD30 bound the *ZmPIN1c* and *ZmVG1* promoter fragments and activated their transcription, whereas ZmIDD31 did not show detectable binding to these fragments in EMSAs. This difference suggests that the two paralogs may have diverged in the regulation of at least some downstream genes despite their similar expression patterns. Nevertheless, because only selected promoter regions were examined, their broader target repertoires and possible functional redundancy remain to be determined.

PIN1‐mediated auxin transport has been implicated in the PLB formation and ligule outgrowth (Johnston et al., 2014; Moon et al., 2013; Neher et al., 2023; Zhong et al., 2025). At the transcriptional level, ZmLG1 directly activates *ZmPIN1a*, *ZmPIN1b*, and *ZmPIN1c* (Zhong et al., 2025). ZmIDD14 and ZmIDD15 act together with ZmILI1 to promote *ZmLG1* expression (Liu et al., 2024), thereby linking these IDD family members indirectly to PIN1 regulation. Our findings identify a different regulatory connection: ZmIDD30 directly binds to the *ZmPIN1c* promoter and activates its transcription. Genetic and spatial analyses will be required to determine whether ZmIDD30 contributes to ligule development through the regulation of *ZmPIN1c* and how this regulatory relationship affects local auxin transport and distribution.

A second potential aspect of ZmIDD30 function involves lipid metabolism. The semi‐dominant *Vg1* mutation causes ligule degeneration through aberrant splicing of a gene encoding a putative cyclopropane fatty acid synthase (Liu, et al., 2019). Enzymes of this class modify unsaturated fatty‐acyl chains in phospholipids, producing cyclopropane fatty acids and thereby altering phospholipid fatty acid composition (Bao et al., 2002; Bao et al., 2003). Together, these findings that ZmIDD30 bound the *ZmVG1* promoter fragment and activated its transcription establish a regulatory link between ZmIDD30 and ZmVG1, suggesting that ZmIDD30 may influence ligule development through ZmVG1‐mediated lipid modification.

In conclusion, our study establishes a single‐nucleus framework for maize ligule development, provides an epidermis‐to‐ligule developmental model, and identifies ZmIDD30 as a candidate regulator associated with both PIN1‐mediated auxin transport and ZmVG1‐related lipid metabolism. Although our sampling captured the PLB formation and ligule outgrowth, additional early developmental stages would further refine the trajectory of ligule specification. Future integration with epigenomic and proteomic data, together with comparative analyses across grass species, should help clarify conserved and lineage‐specific regulatory mechanisms. Genetic analysis of ZmIDD30 and ZmIDD31 will also be important for defining their contributions to ligule development.

## MATERIALS AND METHODS

### Plant materials and growth conditions

Maize (*Zea mays* L.) inbred line B73 was used in this study. Seeds were germinated on moist filter paper in the dark at 25°C. After approximately 2 days, when coleoptiles reached 1–2 mm in length, uniformly germinated seeds were selected and transferred to soil. The plants were grown in a growth chamber under a 16‐h light/8‐h dark photoperiod at 25°C. Separately, plants for protoplast isolation were grown in continuous darkness at 25°C after germination.

### Cryo‐scanning electron microscopy (cryo‐SEM)

To characterize the developmental stages of the maize ligule, the ligular regions from the third and fourth leaves of 12‐day‐old seedlings were harvested, mounted onto brass stubs, and immediately cryofixed by plunging into liquid nitrogen slush at −210°C. Cryofixed samples were transferred to a Quorum PP3010T high‐vacuum cryo‐preparation system (Quorum Technologies, UK). To remove surface frost, the specimen holder was warmed to −95°C for 10–15 min and then rapidly cooled to −140°C. The sample surfaces were then sputter‐coated with a 5–10 nm gold/palladium (Au/Pd) layer. The coated samples were transferred via a cryo‐transfer shuttle onto the cold stage of a Zeiss Sigma 300 field emission scanning electron microscope (Carl Zeiss, Germany). High‐resolution imaging was performed using a secondary electron detector at an accelerating voltage of 7 kV and a working distance of 5–10 mm.

### Sample preparation, library construction, and sequencing

Nuclei were isolated following a previously described protocol (Sun et al., 2022) with minor modifications. Ligular regions from the third and fourth leaves of 12‐day‐old seedlings were separately microdissected on ice (*n* = 30 per sample) and immediately transferred to pre‐chilled Galbraith buffer (Galbraith et al., 1983). The harvested tissues were then transferred to 700 μL Galbraith buffer supplemented with 0.2% (w/v) bovine serum albumin (BSA), 1 mM dithiothreitol (DTT), and 0.4 U/μL RNase inhibitor (Roche) in a plastic Petri dish. The tissues were finely chopped using razor blades on ice to release the nuclei, and the homogenate was filtered through a 40‐μm cell strainer to remove large cellular debris. After staining with 50 μg/mL propidium iodide (PI), nuclei were purified using a Beckman Moflo Astrios EQ cell sorter operated in single‐cell mode with a 70‐μm nozzle, using diethyl pyrocarbonate (DEPC)‐treated phosphate‐buffered saline (PBS) as the sheath fluid. The quality and concentration of the sorted nuclei were assessed using phase‐contrast microscopy (Nikon Eclipse Ts2) and a hemocytometer. Nuclei suspensions used for library construction typically contained more than 70% intact nuclei at concentrations of 600–700 nuclei/μL. Approximately 20 000 sorted nuclei were loaded onto the 10x Genomics Chromium Controller for library construction. Libraries were prepared using the Chromium Next GEM Single Cell 3′ Kit v3.1 according to the manufacturer's instructions and sequenced on an Illumina NovaSeq 6000 platform with 150‐bp paired‐end reads.

### snRNA‐seq pre‐processing, quality control, and clustering

The maize B73 reference genome (v4) and corresponding GTF annotation files were downloaded from Ensembl Plants release 49. Raw snRNA‐seq reads were processed using Cell Ranger (v7.0.1; 10× Genomics) with the ‐‐include‐introns parameter enabled to account for unspliced pre‐mRNA in nuclei. The S2 library was processed with default parameters, whereas the S1 library was processed with ‐‐force‐cells = 12 000 based on the expected number of recovered nuclei. The resulting gene‐barcode matrices were used for all downstream analyses.

The Seurat R package (v4.4.0) (Hao et al., 2021) was utilized for quality control and downstream analyses. Quality control was applied to each sample independently. Based on the distribution of detected gene numbers, nuclei with fewer than 1000 or more than 3000 detected genes were excluded for S1, whereas a threshold of 1000–4500 detected genes was applied to S2. The median proportions of mitochondrial and chloroplast transcripts were low in both samples, with the upper quartiles below 0.23%. Therefore, no additional filtering based on organellar transcript proportions was applied. Following initial clustering, clusters showing uniformly low UMI counts and low detected gene numbers were removed as low‐quality debris‐like clusters. Putative doublets were then computationally identified and removed using DoubletFinder (v2.0.3) (Mcginnis et al., 2019), with the expected doublet rate estimated according to the number of loaded nuclei for each 10× Genomics library.

For dataset integration, each dataset was independently normalized using SCTransform with default parameters. To better capture transcriptional heterogeneity across developmental stages, integration features were selected using SelectIntegrationFeatures (nfeatures = 6000), and the datasets were prepared for integration using PrepSCTIntegration. Integration anchors were identified using the FindIntegrationAnchors function (normalization.method = “SCT”, k.filter = 300), followed by dataset integration using IntegrateData. After integration, principal component analysis (PCA) was performed using RunPCA (npcs = 100), and the top 50 principal components were used to construct the SNN graph with FindNeighbors, followed by clustering with FindClusters at a resolution of 1.0 and UMAP visualization with RunUMAP (n.neighbors = 50, min.dist = 0.5, and n.components = 2).

Differentially expressed genes (DEGs) for each cluster were identified using the FindAllMarkers function in Seurat with the Wilcoxon rank‐sum test. Genes with min.pct ≥0.01, average log_2_ fold change >0, and adjusted *P* value <0.05 were retained as cluster‐enriched genes. Cell identities were manually assigned based on the expression of previously reported marker genes (Table S2).

### Comparison between snRNA‐seq and LM‐RNA‐seq datasets

Lists of ligule‐upregulated genes identified in the all‐cell‐layer and adaxial epidermal samples (373 and 287 genes, respectively) were obtained from the supplementary dataset of a previously published LM‐RNA‐seq study (Johnston et al., 2014) and converted from B73 RefGen_v3 to RefGen_v4 gene model IDs using the Translate Gene Model IDs tool in MaizeGDB (https://www.maizegdb.org/). After conversion and removal of unmapped or duplicate entries, 314 and 235 unique RefGen_v4 genes remained for the all‐cell‐layer and adaxial epidermal datasets, respectively, and these converted gene sets were used for subsequent analyses. The two converted gene lists were independently intersected with the DEGs identified in each snRNA‐seq cluster. The significance of each overlap was assessed using a hypergeometric test, with the union of all cluster‐enriched genes across the snRNA‐seq dataset after removal of duplicates (11 613 unique genes) used as the gene universe, followed by Benjamini‐Hochberg correction for multiple comparisons. Adjusted *P* values below 0.01 and 0.001 were considered significant and highly significant, respectively.

### Gene ontology (GO) enrichment analysis

GO annotations for maize genes were retrieved from agriGO (v2.0) (Tian et al., 2017). GO enrichment analysis was performed using the R package clusterProfiler (v4.10.1) (Wu et al., 2021) for each gene module identified along the pseudotime trajectory. GO terms with a *P* value < 0.05 were considered to be significantly enriched.

### Pseudotime trajectory analysis

Manifold learning was performed on the epidermal lineage clusters, including clusters 0, 4, 9, 12, and 21, using the DDRTree algorithm (Mao et al., 2015). Hyperparameters were set to sigma = 1e‐5, param.gamma = 15, and ncenter = 55 to capture the branching topology. Developmental pseudotime was inferred using Slingshot (Street et al., 2018), with cluster 0 specified as the root because it was located in the shared pre‐bifurcation region of the epidermal trajectory and was relatively enriched in the earlier S1 sample (Figure S3), consistent with a relatively early epidermal state. The trajectory terminating in cluster 9 was interpreted as the ligule branch based on its ligule‐associated marker genes. To model expression kinetics along the trajectory, stochastic noise was reduced via a sliding‐window average (step = 0.08), followed by LOESS regression (span = 0.35, degree = 1). Genes were partitioned into three modules based on hierarchical clustering of fitted expression trends across 80 equidistant pseudotime points.

### Co‐expression network analysis

Prior to network analysis, genes expressed in fewer than 10 cells were excluded from the SCTransform‐normalized expression matrix. The filtered dataset was modeled using the singleCellGGM algorithm (Xu et al., 2024). To ensure edge reliability, gene pairs with a false discovery rate (FDR) below 0.05 were selected to establish the global co‐expression network. These filtered gene pairs were then partitioned into discrete co‐expression modules using the Markov cluster algorithm (MCL, inflation parameter = 1.5). Ligule‐enriched co‐expression modules were subsequently extracted and visualized using Cytoscape (v3.10.3) (Shannon et al., 2003).

Candidate core transcription factors were identified using the MINI‐EX (Ferrari et al., 2022). The maize transcription factor list and binding motif information were obtained from PlantTFDB v5.0.

### Fluorescence *in situ* hybridization (FISH)

Shoot apices approximately 1 cm in length were collected from 10‐day‐old maize seedlings after removing the coleoptiles and the two outermost leaves. Samples were immediately fixed overnight in RNase‐free FAA fixative. Paraffin‐embedded tissues were sectioned at a thickness of 4 μm. To verify shoot apex morphology, adjacent sections were pre‐screened using toluidine blue O (TBO) staining before FISH. Sections were deparaffinized, rehydrated, and pretreated with 3% H_2_O_2_ in methanol for 10 min. After washing with DEPC‐treated water, sections were incubated in 0.2 M HCl for 15 min and then digested with Proteinase K at 37°C for 20 min. Digestion was terminated with 0.1 M glycine, followed by post‐fixation in 4% paraformaldehyde for 10 min. To reduce non‐specific background, sections were acetylated with acetic anhydride (pH 8.0) and rinsed with 5x SSC (pH 7.5). Cy5‐labeled antisense and sense probes for ZmIDD30 (sequences listed in Table S8) were synthesized by Gefan Biotechnology (Shanghai, China), with the sense probe serving as the negative control. Pre‐hybridization was performed at 65°C for 1 h, followed by hybridization in the dark at 65°C for 48 h using 500 ng/mL of the respective Cy5‐labeled probe. After hybridization, sections were subjected to three stringent washes for 10 min each in a 1:1 mixture of formamide and 4x SSC (pH 4.5) at 60°C. Finally, sections were counterstained with DAPI, mounted, and imaged using a Nikon Eclipse Ci fluorescence microscope.

### DNA and RNA extraction, cDNA synthesis, and plasmid construction

Total RNA was extracted from whole maize seedlings using TRIzol reagent (Invitrogen, USA) and reverse‐transcribed using the PrimeScript RT Reagent Kit with gDNA Eraser (RR047A, Takara, Shiga, Japan). Genomic DNA was isolated using the CTAB method. For plasmid construction, the full‐length coding sequences (CDSs) of *ZmIDD30* and *ZmIDD31* were amplified from cDNA, whereas the approximately 2‐kb promoter regions of *ZmVG1* and *ZmPIN1c* were amplified from genomic DNA using high‐fidelity DNA polymerase (RR371A, Takara, Shiga, Japan). All PCR products were cloned into linearized vectors using the ClonExpress II One Step Cloning Kit (C112, Vazyme, Nanjing, China). Specifically, the *ZmIDD30* and *ZmIDD31* CDSs were inserted in‐frame into *EcoRI*‐linearized *pGreen II 62‐SK‐GFP* for subcellular localization analysis; the *ZmIDD30* and *ZmIDD31* CDSs were cloned into *EcoRI*‐linearized *pGEX‐4 T‐1* for GST‐fusion protein expression; and promoter fragments of *ZmVG1* and *ZmPIN1c* were inserted into *XhoI*‐linearized *pGreen II 0800‐LUC* for luciferase reporter assays. All constructed vectors were verified by PCR and Sanger sequencing. Primers used are listed in Table S8.

### CUT&Tag assays

To identify ZmIDD30‐associated DNA‐binding regions, CUT&Tag assays were performed using transiently transformed maize protoplasts. Maize protoplasts were isolated from the second leaves of 14‐day‐old etiolated seedlings and transiently transformed as previously described with minor modifications (Gao et al., 2019; Ma et al., 2022). Briefly, leaf strips were enzymatically digested to release protoplasts, which were then purified, resuspended in MMG buffer, and adjusted to 2 × 10^6^ cells/mL. Protoplast viability was assessed by FDA and PI staining, and only preparations with viability greater than 95% were used for transformation. For each transformation, 50 μg of GFP control or ZmIDD30‐GFP plasmid was introduced into 1 × 10^6^ protoplasts using PEG‐mediated transformation. Transformed protoplasts were cultured in the dark at 25°C for 18 h, and transformation efficiency was evaluated based on GFP fluorescence. Three independent technical replicates were performed for each group.

CUT&Tag libraries were prepared using the Hyperactive Universal CUT&Tag Assay Kit for Illumina Pro (TD904, Vazyme, Nanjing, China) according to the manufacturer's instructions. Briefly, transformed protoplasts were immobilized on pre‐activated ConA Beads Pro and incubated overnight at 4°C with rabbit anti‐GFP antibody (1:100, P30010, Abmart, Shanghai, China), followed by incubation with goat anti‐rabbit IgG antibody (1:100, Ab207, Vazyme). The samples were then treated with pA/G‐Tn5 transposase, subjected to tagmentation, and DNA fragments were extracted, PCR‐amplified, purified, and sequenced on an Illumina NovaSeq 6000 platform.

Clean reads were obtained by quality filtering using fastp (v1.0) (Chen, 2025) and adapter trimming using Trim Galore (v0.6.10) (Krueger et al., 2023). The filtered reads were mapped to the maize B73 v4 reference genome from MaizeGDB using Bowtie2 (v2.5.4) (Langmead & Salzberg, 2012). Aligned reads were sorted and indexed using SAMtools (v1.10) (Danecek et al., 2021). Peak calling was performed using MACS2 (v2.2.7.1) (Zhang et al., 2008) by comparing ZmIDD30‐GFP with the GFP control, with a significance cutoff of *q* < 0.05. Motif analysis was performed on the merged peak regions using HOMER with the maize reference genome (Heinz et al., 2010). The selected motif matrices were then used by FIMO to locate motif occurrences within the 3‐kb promoter regions of ligule‐related genes, with ‐‐thresh 1e‐4.

### Electrophoretic mobility shift assays (EMSAs)

The GST‐ZmIDD30 and GST‐ZmIDD31 fusion proteins were expressed in *Escherichia coli* BL21 (DE3) by induction with 0.5 mM isopropyl β‐D‐1‐thiogalactopyranoside (IPTG) at 18°C for 20 h and purified using BeyoGold™ GST‐tag Purification Resin (P2251, Beyotime, Shanghai, China). For probe preparation, complementary 40‐bp oligonucleotides derived from the *ZmLG1*, *ZmVG1*, and *ZmPIN1c* promoters were synthesized by Generay Biotechnology (Shanghai, China) (Table S8). Double‐stranded probes were generated by annealing complementary strands at 95°C for 5 min, followed by gradual cooling to room temperature. The wild‐type probes were biotin‐labeled at the 5′ end, whereas unlabeled wild‐type and mutant probes served as specific and mutant competitors, respectively.

EMSAs were performed using a Chemiluminescent EMSA Kit (GS009, Beyotime, Shanghai, China) according to the manufacturer's instructions. Briefly, for the binding assay, purified GST‐ZmIDD30 and GST‐ZmIDD31 were separately incubated with unlabeled wild‐type competitors (10‐fold, 50‐fold) or mutant competitors (10‐fold) at room temperature, followed by the addition of biotin‐labeled probes. Purified GST protein was used as a negative control. DNA‐protein complexes were separated on native polyacrylamide gels and detected by chemiluminescence.

### Transient luciferase reporter assays

For transcriptional activity assays, the recombinant constructs were transformed into *Agrobacterium tumefaciens* strain GV3101 carrying pSoup‐p19. Individual *Agrobacterium* cultures were resuspended in infiltration buffer containing 10 mM MES (pH 5.6) and 10 mM MgCl_2_ to an OD_600_ of 1.0. For co‐infiltration, the reporter culture (*proZmVG1::LUC or proZmPIN1c::LUC*) was mixed with either the control (*GFP*) or effector culture (*ZmIDD30*) at a 2:1 (v/v) ratio. After supplementation with 200 μM acetosyringone and incubation at room temperature for 30 min, the mixtures were infiltrated into leaves of 4‐week‐old *Nicotiana benthamiana* plants. After 48 h, bioluminescence was induced by applying 1 mM D‐luciferin onto the leaves. Following incubation in the dark for 5 min, luminescence signals were recorded using a Tanon 5200 Multi Imaging System (Shanghai, China). The intensity of LUC signals was quantified using TanonImage software. Data from three independent biological replicates were analyzed, and statistical significance between groups was determined using Student's *t*‐test.

## AUTHOR CONTRIBUTIONS

GS, JP, and XY designed and supervised the project. FW and JP prepared materials for the snRNA‐seq and CUT&Tag assay. FW and MX analyzed the data. FW performed the functional assays and wrote the original draft. GS, JP, XY, DWG, JH, and C‐PS revised the manuscript. All authors reviewed and approved the final manuscript.

## CONFLICT OF INTEREST

The authors declare that they have no competing interests.

## Supporting information


**Figure S1.** Nuclei sorting and quality assessment for snRNA‐seq samples from the maize ligular region. (a–e) S1 sample, representing the preligule band formation stage. (f–j) S2 sample, representing the ligule outgrowth stage. (a, f) Nuclei were first gated according to forward scatter height (FSC‐H) and side scatter area (SSC‐A) to enrich nuclei and reduce low‐scatter debris. (b, g) Singlet nuclei were selected based on the relationship between FSC‐H and FSC‐A to reduce aggregates. (c, h) PI‐positive nuclei were further selected based on propidium iodide area (PI‐A) fluorescence and FSC‐A. (d, i) PI‐A histograms displaying DNA‐content peaks corresponding to 2C and 4C nuclei used for downstream library preparation. (e, j) Sorted nuclei were examined by phase‐contrast microscopy to confirm nuclear integrity. Scale bars: 20 μm.
**Figure S2**. Post‐sequencing quality assessment of the gene‐barcode expression matrix. (a, b) Distributions of unique molecular identifiers (UMIs) per nucleus (a) and detected genes per nucleus (b) before filtering. (c, d) Distributions of UMIs per nucleus (c) and detected genes per nucleus (d) for nuclei retained after filtering. (e, f) UMAP plots showing singlets and predicted doublets in S1 (e) and S2 (f). (g, h) Proportions of mitochondrial transcripts (g) and chloroplast transcripts (h) in the retained nuclei.
**Figure S3**. Nuclei counts across integrated clusters in the snRNA‐seq dataset of the maize ligular region. Numbers above each bar indicate nuclei counts from S1 or S2 in the corresponding integrated cluster.
**Figure S4**. Expression patterns of marker genes used to annotate non‐ligule cell populations in the maize ligular‐region snRNA‐seq dataset. Feature plots showing the distribution of representative marker gene expression across the integrated UMAP embedding. Panels show marker genes for epidermis (a), stomatal lineage (b, c), phloem (d), xylem (e), bundle sheath (f), mesophyll (g), S phase (h), and G2/M phase (i).
**Figure S5**. Expression patterns of genes previously implicated in maize ligule development in the ligular‐region snRNA‐seq dataset. (a) Dot plot showing the expression of ligule‐related genes reported in previous studies across clusters 0–22. Dot size indicates the percentage of nuclei expressing each gene, and dot color indicates the average normalized expression level within each cluster. (b) Feature plots showing the expression distribution of representative ligule‐related genes (*ZmLG1*, *ZmIDD31*, and *ZmVG1*) across all nuclei. Color intensity reflects normalized expression level.
**Figure S6**. Expression of *ZmIDD30* and *ZmIDD31* in the laser microdissection RNA sequencing (LM‐RNA‐seq) dataset. Bar plots show counts per million (CPM) values of *ZmIDD30* and *ZmIDD31* across the ligule, blade, and sheath based on published LM‐RNA‐seq data (Johnston et al., 2014). (a, c) CPM values from all‐cell‐layer samples for *ZmIDD30* (a) and *ZmIDD31* (c). (b, d) CPM values from adaxial epidermis samples for *ZmIDD30* (b) and *ZmIDD31* (d). Dots indicate individual biological replicates, and *P* values for pairwise comparisons are shown above the bars.
**Figure S7**. Expression patterns of ligule‐ and stomata‐associated marker genes along the developmental trajectory. Feature plots showing the expression distribution of representative ligule‐associated genes (a) and stomata‐associated genes (b), including *ZmLG1*, *ZmIDD31*, *ZmVG1*, *ZmTMM1*, *ZmMUTE*, and *ZmFAMA*, across the reconstructed epidermal trajectory. Color intensity indicates normalized expression levels.
**Figure S8**. Stage‐associated expression patterns of cytokinin‐ and brassinosteroid‐related genes in the ligule population. Bar plots show the average normalized expression levels of genes associated with cytokinin metabolism and signaling (a) and brassinosteroid signaling (b) in S1‐ and S2‐derived cells of the ligule population.
**Figure S9**. Expression patterns and pseudotemporal profiles of representative genes in the M27 and M06 co‐expression modules. (a–c) Feature plots and pseudotime expression profiles of representative M27 genes, including *ZmIAL1* (a), *ZmBOP1b* (b), and *ZmIDD28* (c). Feature plots show gene expression in S1 and S2, and pseudotime plots show expression dynamics along the epidermis‐to‐ligule trajectory. Dots represent individual nuclei, and solid lines indicate LOESS‐fitted expression trends. (d‐g) Feature plots showing the expression of representative M06 genes in S1 and S2, including *ZmIDD30* (d), *ZmVG1* (e), *ZmLG1* (f), and *ZmPIN1c* (g).
**Figure S10**. Quality assessment and reproducibility of the ZmIDD30 CUT&Tag data. (a) Pearson correlation heatmap of genome‐wide CUT&Tag signals across three technical replicates of ZmIDD30 and GFP control. Values indicate pairwise Pearson correlation coefficients. (b, c) Strand cross‐correlation profiles of the GFP control (b) and ZmIDD30 CUT&Tag libraries (c). Red dashed lines indicate the predominant fragment lengths inferred from the strand cross‐correlation peaks. NSC, normalized strand cross‐correlation coefficient; RSC, relative strand cross‐correlation coefficient. Qtag indicates the library‐quality classification derived from strand cross‐correlation analysis.
**Figure S11**. EMSA assays testing ZmIDD30 binding to candidate promoter motifs. EMSAs were performed using probes containing two Motif 1 elements from the *ZmLG1* promoter (a) and a Motif 2 element from the *ZmPIN1c* promoter (b). The position of each tested motif relative to the translational start site (ATG) is shown above the corresponding gel. The probe 1 motif in the *ZmLG1* promoter is located on the antisense strand. Competition assays were performed using 10‐ and 50‐fold molar excesses of unlabeled wild‐type probes and a 10‐fold molar excess of the corresponding mutant probes, as indicated. GST alone was used as a negative control.
**Figure S12**. Purification profiles of recombinant GST‐ZmIDD30 and GST‐ZmIDD31 proteins. SDS‐PAGE analysis showing the expression and purification of GST‐ZmIDD30 (a) and GST‐ZmIDD31 (b). Lanes 1 and 2 show total proteins before and after IPTG induction, respectively, followed by successive L‐GSH elution fractions (lanes 3–6 in (a) and lanes 3–5 in (b)). The expected recombinant protein bands are indicated.
**Figure S13**. Subcellular localization and EMSA analysis of ZmIDD31. (a) Subcellular localization of ZmIDD31‐GFP in maize protoplasts. Scale bars: 10 μm. (b) Pseudotime expression pattern of *ZmIDD31* along the epidermis‐to‐ligule differentiation trajectory. Dots represent individual nuclei, and the line represents the LOESS‐fitted expression trend. (c‐e) EMSA assays testing the binding of ZmIDD31 to motif‐containing probes from the *ZmVG1* (c), *ZmPIN1c* (d) and *ZmLG1* (e) promoters. Biotin‐labeled probes were incubated with purified GST‐ZmIDD31 protein, with GST protein used as a negative control.

**Table S1.** Summary statistics for the maize ligule snRNA‐seq datasets before and after quality control.
**Table S2**. Marker genes used for cell‐type annotation in the snRNA‐seq dataset of the maize ligular region.
**Table S3**. Cluster‐enriched genes identified in the maize ligular‐region snRNA‐seq dataset.
**Table S4**. Enrichment of ligule‐upregulated genes identified by LM‐RNA‐seq among cluster‐specific differentially expressed genes in the snRNA‐seq dataset of the ligular region.
**Table S5**. Gene Ontology biological process enrichment analysis of gene modules M1‐M3 identified along the inferred ligule trajectory.
**Table S6**. Genes assigned to the stage‐biased co‐expression modules M06 and M27.
**Table S7**. Top 10 motifs enriched in ZmIDD30 target sequences and their HOMER‐based annotations.
**Table S8**. Primers and oligonucleotide probes used in this study.

## Data Availability

The raw snRNA‐seq and CUT&Tag sequencing data have been deposited in ScienceDB under https://doi.org/10.57760/sciencedb.44503 and https://doi.org/10.57760/sciencedb.44720, respectively.
