# Supplementary material for: Single‐nucleus transcriptomics reveals transcriptional dynamics underlying maize ligule development
Source: Plant J. 2026 Nov 9;128(3):e71194. doi: 10.1111/tpj.71194 (PMC13748927; doi:10.1111/tpj.71194)
Supplement: Supplementary file 1 [file TPJ-128-0-s002.docx]

**Figure S1. Nuclei sorting and quality assessment for snRNA-seq samples from the maize ligular region.** (a-e) S1 sample, representing the preligule band formation stage. (f-j) S2 sample, representing the ligule outgrowth stage. (a, f) Nuclei were first gated according to forward scatter height (FSC-H) and side scatter area (SSC-A) to enrich nuclei and reduce low-scatter debris. (b, g) Singlet nuclei were selected based on the relationship between FSC-H and FSC-A to reduce aggregates. (c, h) PI-positive nuclei were further selected based on propidium iodide area (PI-A) fluorescence and FSC-A. (d, i) PI-A histograms displaying DNA-content peaks corresponding to 2C and 4C nuclei used for downstream library preparation. (e, j) Sorted nuclei were examined by phase-contrast microscopy to confirm nuclear integrity. Scale bars: 20 μm.

**Figure S2. Post-sequencing quality assessment of the gene-barcode expression matrix.** (a, b) Distributions of unique molecular identifiers (UMIs) per nucleus (a) and detected genes per nucleus (b) before filtering. (c, d) Distributions of UMIs per nucleus (c) and detected genes per nucleus (d) for nuclei retained after filtering. (e, f) UMAP plots showing singlets and predicted doublets in S1 (e) and S2 (f). (g, h) Proportions of mitochondrial transcripts (g) and chloroplast transcripts (h) in the retained nuclei.

**Figure S3. Nuclei counts across integrated clusters in the snRNA-seq dataset of the maize ligular region.** Numbers above each bar indicate nuclei counts from S1 or S2 in the corresponding integrated cluster.

**Figure S4. Expression patterns of marker genes used to annotate non-ligule cell populations in the maize ligular-region snRNA-seq dataset.** Feature plots showing the distribution of representative marker gene expression across the integrated UMAP embedding. Panels show marker genes for epidermis (a), stomatal lineage (b,c), phloem (d), xylem (e), bundle sheath (f), mesophyll (g), S phase (h), and G2/M phase (i).

**Figure S5. Expression patterns of genes previously implicated in maize ligule development in the ligular-region snRNA-seq dataset.** (a) Dot plot showing the expression of ligule-related genes reported in previous studies across clusters 0-22. Dot size indicates the percentage of nuclei expressing each gene, and dot color indicates the average normalized expression level within each cluster. (b) Feature plots showing the expression distribution of representative ligule-related genes (*ZmLG1*, *ZmIDD31*, and *ZmVG1*) across all nuclei. Color intensity reflects normalized expression level.

**Figure S6. Expression of *ZmIDD30* and *ZmIDD31* in the laser microdissection RNA sequencing (LM-RNA-seq) dataset.** Bar plots show counts per million (CPM) values of *ZmIDD30* and *ZmIDD31* across the ligule, blade, and sheath based on published LM-RNA-seq data (Johnston et al., 2014). (a, c) CPM values from all-cell-layer samples for *ZmIDD30* (a) and *ZmIDD31* (c). (b, d) CPM values from adaxial epidermis samples for *ZmIDD30* (b) and *ZmIDD31* (d). Dots indicate individual biological replicates, and *P* values for pairwise comparisons are shown above the bars.

**Figure S7. Expression patterns of ligule- and stomata-associated marker genes along the developmental trajectory.** Feature plots showing the expression distribution of representative ligule-associated genes (a) and stomata-associated genes (b), including *ZmLG1*, *ZmIDD31*, *ZmVG1*, *ZmTMM1*, *ZmMUTE*, and *ZmFAMA*, across the reconstructed epidermal trajectory. Color intensity indicates normalized expression levels.

**Figure S8. Stage-associated expression patterns of cytokinin- and brassinosteroid-related genes in the ligule population.** Bar plots show the average normalized expression levels of genes associated with cytokinin metabolism and signaling (a) and brassinosteroid signaling (b) in S1- and S2-derived cells of the ligule population.

**Figure S9. Expression patterns and pseudotemporal profiles of representative genes in the M27 and M06 co-expression modules.** (a-c) Feature plots and pseudotime expression profiles of representative M27 genes, including ZmIAL1 (a), ZmBOP1b (b), and ZmIDD28 (c). Feature plots show gene expression in S1 and S2, and pseudotime plots show expression dynamics along the epidermis-to-ligule trajectory. Dots represent individual nuclei, and solid lines indicate LOESS-fitted expression trends. (d-g) Feature plots showing the expression of representative M06 genes in S1 and S2, including ZmIDD30 (d), ZmVG1 (e), ZmLG1 (f), and ZmPIN1c (g).

**Figure S10. Quality assessment and reproducibility of the ZmIDD30 CUT&Tag data.** (a) Pearson correlation heatmap of genome-wide CUT&Tag signals across three technical replicates of ZmIDD30 and GFP control. Values indicate pairwise Pearson correlation coefficients. (b, c) Strand cross-correlation profiles of the GFP control (b) and ZmIDD30 CUT&Tag libraries (c). Red dashed lines indicate the predominant fragment lengths inferred from the strand cross-correlation peaks. NSC, normalized strand cross-correlation coefficient; RSC, relative strand cross-correlation coefficient. Qtag indicates the library-quality classification derived from strand cross-correlation analysis.

**Figure S11. EMSA assays testing ZmIDD30 binding to candidate promoter motifs.** EMSAs were performed using probes containing two Motif 1 elements from the *ZmLG1* promoter (a) and a Motif 2 element from the *ZmPIN1c* promoter (b). The position of each tested motif relative to the translational start site (ATG) is shown above the corresponding gel. The probe 1 motif in the *ZmLG1* promoter is located on the antisense strand. Competition assays were performed using 10- and 50-fold molar excesses of unlabeled wild-type probes and a 10-fold molar excess of the corresponding mutant probes, as indicated. GST alone was used as a negative control.

**Figure S12. Purification profiles of recombinant GST-ZmIDD30 and GST-ZmIDD31 proteins.** SDS-PAGE analysis showing the expression and purification of GST-ZmIDD30 (a) and GST-ZmIDD31 (b). Lanes 1 and 2 show total proteins before and after IPTG induction, respectively, followed by successive L-GSH elution fractions (lanes 3-6 in (a) and lanes 3-5 in (b)). The expected recombinant protein bands are indicated.

**Figure S13. Subcellular localization and EMSA analysis of ZmIDD31.** (a) Subcellular localization of ZmIDD31-GFP in maize protoplasts. Scale bars: 10 μm. (b) Pseudotime expression pattern of *ZmIDD31* along the epidermis-to-ligule differentiation trajectory. Dots represent individual nuclei, and the line represents the LOESS-fitted expression trend. (c-e) EMSA assays testing the binding of ZmIDD31 to motif-containing probes from the *ZmVG1* (c), *ZmPIN1c* (d) and *ZmLG1* (e) promoters. Biotin-labeled probes were incubated with purified GST-ZmIDD31 protein, with GST protein used as a negative control.
